# The Longitudinal Examination of Habitual Sleep Duration in Relation to Weight Gain Risk Behaviors and Body Composition Changes Among College Students: Project XXXXXXX

**DOI:** 10.21203/rs.3.rs-6771202/v1

**Published:** 2025-06-09

**Authors:** Andrea T. Kozak, Matthew Parker Lico, Nicole L. Jarrett, Scott M. Pickett

**Affiliations:** Oakland University; Oakland University; Oakland University; Florida State University

**Keywords:** sleep duration, sleep quality, obesity risk, college students

## Abstract

**Background:**

The purpose of the current study was to examine habitual sleep duration trajectories across the first two years of college and determine whether the trajectories were related to weight gain risk behaviors and increases in body mass index (BMI) and percent body fat.

**Methods:**

A sample of 115 students with a BMI between 18.5–29.9 were enrolled prior to beginning their first year of college. Data were collected at three time points across the first two years of college and data collection was conducted both in laboratory and remotely. Participants participated in a total of eight in-person sessions and three eight-day, at-home recording periods. There were objective measures of body fat composition (i.e., dual-energy X-ray absorptiometry), BMI, sleep (i.e., Phillips Respironics Actiwatch 2 Device), and physical activity (i.e., Actigraph wGT3x-BT accelerometer). Self-report measures of sleep quality (i.e., Pittsburgh Sleep Quality Index) and food and beverage intake (i.e., ASA-24) were also collected.

**Results:**

The findings suggest that there were no habitual sleep duration trajectories observed; therefore, none of outcome variables were examined in relation to trajectories. Follow-up mixed effects models suggest that as poor sleep quality worsened, BMI increased both within participants and across the sample. No other significant relationships were found between sleep duration and quality and the other outcome variables.

**Conclusions:**

The findings highlight subjective sleep quality as potential key component in relation to obesity-related changes. Sample characteristics may have also played a role in the limited findings between the sleep variables and the other obesity-risk outcome variables.

## Introduction

College is the time in which independence from parents/caregivers is established, setting the stage for future health behavior choices such as frequency of daily physical activity. Unfortunately, an estimated 30% of the almost 19,000 U.S. college students surveyed by the American College Health Association [1] do not meet the suggested recommendations regarding aerobic activity. Additionally, when the guidelines for both aerobic activity and strength training are taken into account, approximately 59% of these U.S. college students do not meet these recommendations. The situation regarding eating is not any better as approximately 85% of U.S. college students reportedly do not eat at least 3 servings of fruit and 74% refrain from consuming at least 3 servings of vegetables. In one longitudinal study, half of 204 freshmen college students surveyed ate fast foods high in fat at least two times per week, and this behavior was still apparent in their senior year [2]. Another longitudinal study found that as adolescents transitioned to young adulthood, they frequently avoided breakfast and ate more unhealthy snacks [3]. In sum, a majority of young adults do not consume a high-quality diet, which means their dietary intake does not align with dietary guidelines [4].

Physical inactivity and unhealthy eating are important reasons for why just under 33% of college-aged adults reportedly have a body weight that places them in the category of obesity [5]. Young adults in the category of overweight or obesity are at a significant risk of moving to an even higher BMI category in comparison to any other age group in adulthood [6]. A higher BMI in young adulthood is associated with poor health-related quality of life in middle age [7]. Simply gaining a ½ pound to 1 pound each year starting in young adulthood places people at an increased risk of premature death due to CVD or cancer later on [8].

Unhealthy eating and physical inactivity have consistently captured the attention of researchers and the public alike given that reduced caloric consumption and increased physical activity on a daily basis are both essential for weight loss. Further, in order to maintain a healthy body weight, there needs to be a balance between caloric intake and caloric expenditure via physical activity. However, other factors likely play an important role in placing an individual at risk of gaining weight to place them at the level of overweight or obesity. Inadequate sleep via short sleep duration is a noteworthy factor to examine given its direct and indirect links with overweight and obesity. *Habitual short sleep* occurs when individuals chronically sleep <7 hours a night [9]. A recent meta-analysis of cohort studies focused on adults found that habitual short sleep duration was significantly associated with obesity [10]. There is also evidence from experimental and cohort studies with children and adolescents establishing a link between habitual short sleep duration and excess weight at the overweight or obese level [11]. It is important to note that many studies have relied upon subjective measures of sleep duration given logistical and cost issues.

There is also some evidence linking habitual short sleep to unhealthy behavioral choices in adolescents. For example, habitual short sleep (defined as <6 hours of sleep each night) was significantly related to a lower likelihood of eating breakfast daily, eating vegetables, and being physically active at least 3 days a week in a sample of over 2000 high school students [12]. High school students with habitual short sleep were also significantly more likely to consume unhealthy beverages (e.g., sports/energy drinks, sugar-sweetened beverages, diet beverages) compared to those with a longer sleep duration [12]. Experimental studies with adults have found that inducing short sleep is significantly associated with increased caloric intake [13]. Most of the literature to date has focused on sleep duration. However, subjective poor sleep quality is another aspect of inadequate sleep, and this variable has frequently been measured using the Pittsburgh Sleep Quality Index [14]. Fatima and colleagues [14] conducted a meta-analysis of primarily cross-sectional studies with over 25,000 children, adolescents, and young adults and found that poor sleep quality was significantly linked to overweight and obesity. It is noteworthy that most studies in this meta-analysis utilized body mass index (BMI) as the measure of overweight/obesity. The routine use of BMI is not surprising given that it is a very low cost and an easy to calculate option. However, inaccurate information can result from self-reported weight and height and BMI is only an *estimate* of body fat. On the other hand, Vgontzas and colleagues [15] conducted a longitudinal study and found the relationship between subjective short sleep duration and incident obesity was no longer significant among their sample of 815 adults when they controlled for subjective poor sleep quality and emotional stress. This study highlights the role subjective complaints or stressors may have the relationship between sleep duration and obesity risk/obesity.

There is still much to learn about the relationship between inadequate sleep and overweight/obesity in university students. Project XXXXXXX was an observational study designed to address methodological limitations of previous studies. Therefore, longitudinal data were collected with incoming Freshmen university students using objective assessment tools to measure sleep, body fat, and physical activity. Body mass index was assessed on the basis of measured weight and height. Food and beverage consumption always rely on subjective measurement, but a dietary assessment tool, considered to be valid and feasible, developed by the National Cancer Institute was used to collect these data [16]. Project XXXXXXX had two aims: 1) Characterize trajectories of habitual sleep across time, and 2) determine if the identified trajectories of habitual sleep across time are related to weight gain risk behaviors, increases in BMI, and increases in percent body fat. It was hypothesized that different habitual sleep trajectories would be observed across the multiple time points. It was also hypothesized that trajectories indicative of habitual short sleep problems would be associated with higher daily caloric intake on average, lower daily physical activity on average, and increases in BMI and percent body fat.

## Methods

Specific details regarding the methods of Project XXXXXXX can be found in the published protocol paper [17]. Project XXXXXXX was approved by the Institutional Review Board at XXXXXXX (IRB# XXXXXXX) and is registered as a longitudinal observational study on clinicaltrials.gov (XXXXXXX).

### Recruitment and Eligibility

Participants were first year university students with a measured BMI of 18.5–29.9. The minimum age to participate was 17 years old with signed parental permission; participants who were 18 or older did not need a parent’s permission. Participants were ineligible if they were pregnant; not ambulatory; taking a medication that had the possibility of influencing or interfering with sleep; had a past neurological problem, head injury, and/or sleep disorder; or had a current medical or mental health problem/disorder (neurological problem, head injury, sleep disorder, psychosis, mood disorder, anxiety disorder, substance use disorder, suicidal ideation or plans). Participants were recruited via freshmen orientation sessions during the summers of 2017 (Cohort 1) and 2018 (Cohort 2). Names of new students who expressed an interest in participating in psychology-related research were entered into a database and names were randomly drawn in order to extend an invitation to join Project XXXXXXX.

### Procedures

Eligible participants were asked to attend a total of eight individual in-person sessions over a period of two years: one screening/initial training session (Session 1), three booster training sessions (Sessions 3, 5, 7) and four assessment sessions (Sessions 2, 4, 6, 8). During four separate periods over the course of two years, participants completed eight consecutive days of at-home data collection. Eligible participants who attended all eight individual sessions and completed at least five out of eight recording days for each recording period received a total of $140. Participants who moved away from the university were entitled to an additional $25 for travel-related costs for each session attended. In order to discourage withdrawal from the study, participants were compensated immediately after completing relevant tasks.

There were two parts to Session 1: screening and training. During screening, the informed consent process occurred after a photo ID was used to verify age. Potential participants were asked to read the consent form in full and their questions were answered by a research assistant. They signed two copies of the consent form (one was taken home) if they were interested in going forward with participating. Potential participants who were 17 years old received the consent form early and a signature by a parent was required prior to attending session 1. Then a screening interview was conducted, followed by the administration of the adult version of the DSM-5 Self-Rated Level 1 Cross-Cutting Symptom Measure [18] with any relevant DSM-5 Self-Rated Level 2 measures [19]. Weight and height were measured next and then female participants were asked to complete a urine pregnancy test. All eligible participants completed training which consisted of learning portion size estimation [20] and receiving instructions for entering food and beverages into the ASA24, wearing and caring for the sleep and activity monitors, and entering required information (e.g., substances used and medications taken) in a daily diary. Participants also received booster training for all of these activities prior to each eight consecutive days of recording.

During each period of eight consecutive home recording days, participants wore the Actiwatch and Actigraph monitors 24 hours a day, except for when they were bathing or swimming, and entered in the ASA24 all food and beverages they consumed each day. After each period of home recording, participants completed a lab session. The first lab session was after 8 days of recording (Session 2/Time 1), then at 8 months (Session 4/Time 2), at 16 months (Session 6/Time 3), and finally at 24 months (Session 8/Time 4). Lab sessions involved 1) downloading data from the Actigraph and Actiwatch devices, 2) collecting and reviewing the daily diaries, 3) measuring weight and height, 4) completing the PSQI, and 5) being scanned with the Hologic Discovery A dual energy X-ray absorptiometry (DXA) scanner. Only females with a negative pregnancy test had a DXA scan. The current paper reports findings from Time 1-Time 3 due to the COVID-19 pandemic as in-person data collection had to shift to online and behaviors during this time were likely not comparable to past behaviors.

### Measures

#### Demographics and Health.

The researchers created a demographics questionnaire and health questionnaire that participants completed during Session 2. The demographics questionnaire allowed participants to provide information such as race, ethnicity, current residence, and employment status. The health questionnaire contained a comprehensive list of 47 current medical conditions.

#### Objective Measurement of Body Fat.

Participants wore a gown and were asked to lie flat on the dual-energy X-ray absorptiometry (DXA) table during sessions 2, 4, and 6. The technician positioned the participant’s body for a whole-body scan and they were asked to remain still 3 minutes while the scan was completed. The PI of the study completed a 2-day HOLOGIC QDR for Windows Application Training and subsequently trained and supervised technicians. Total body fat was the variable of interest for this study.

#### Body Mass Index.

During sessions 2, 4, and 6, participants’ weight and height were measured without shoes using a digital balance beam scale (Cardinal Detecto Digital Physician’s Scale, Model #6449). The following formula was used to calculate body mass index (BMI), a variable of interest for this study: weight in pounds/(height in inches)^2^ × 703.

#### Objective Measurement of Sleep and Physical Activity.

Sleep duration was measured using the Phillips Respironics Actiwatch 2 Device. The Actiwatch program was utilized for downloading and scoring of the sleep duration data. The sleep data automatically scored by the program was rescored using a standard scoring protocol comparing the Actiwatch scoring with self-reported sleep diary data and adjusting sleep/wake times according to the protocol [21]. In cases where misalignment occurred between self-reported sleep diary and Actiwatch data, the researchers used the Actiwatch data as the guideline per the scoring protocol. Filters were created for each participant at each timepoint to capture only 7 days of sleep. The data were then exported and uploaded to excel for future analysis.

Moderate- and vigorous-intensity minutes of physical activity were assessed with the Actigraph wGT3x-BT accelerometer. The ActiLife program (Version 6.11.9) was utilized for downloading and scoring of the physical activity data (60 second Epoch length) using the Troiano algorithm. If participants completed more than a week of data at a session, scoring was confined to the first 7 days for both sleep and physical activity.

#### Subjective Sleep Quality.

The Pittsburg Sleep Quality Index (PSQI; [22]) is a 19-item self-report measure of sleep quality and it was completed during Sessions 2, 4, and 6. All 19 items are rated on a scale from 0 to 3. Although there are seven “Component” scores (subjective sleep quality, sleep latency, sleep duration, habitual sleep efficiency, sleep disturbances, use of sleep medication, and daytime dysfunction), the overall sleep quality score was used as a variable for this study. The overall sleep quality score ranges from 0–21 and is derived from adding together the seven component scores. Higher scores indicate poorer sleep quality.

#### Measurement of Food and Beverages.

Daily food and beverages were assessed using the that National Cancer Institute’s Automated Self-Administered 24-hour (ASA24) Dietary Assessment Tool [16] in order to obtain the variable of interest, daily kcal, which was averaged over seven days of data for each recording period. Participants were asked to enter their food and beverages all at once before midnight.

### Participants

A total of 116 individuals participated in this study. After preparing the data, one participant was dropped from the analysis due to their sleep duration being greater than three standard deviations from the mean. Table 1 displays detailed information regarding demographic characteristics for 115 participants. Most participants were 18 years old and the average BMI was 23.7. A large number of participants were female. A majority of participants were White. Slightly more participants lived off campus with either parents/caregivers or roommates. Also, a higher percentage of participants were currently working compared to not working. Participants reported the following current medical conditions: 17 had asthma, 13 had migraine headaches, 2 had anemia, 2 had chronic pain, 2 had hypoglycemia, 1 had celiac disease, and 1 had high cholesterol. Overall, the sample was healthy with only 2 participants (1.7%) having 3 medical conditions, 4 participants (3.5%) having 2 medical conditions, and 24 participants (17.4%) having only 1 medical condition.

## Discussion

The primary purpose of Project XXXXXXX was to address methodological limitations of previous studies and use an objective measure of sleep to determine sleep duration trajectories over the first 2 years of college among students. Secondarily, it was expected that the sleep trajectories would be utilized to examine weight gain risk behaviors and changes in BMI and body fat. Specifically, we examined the relationships that different sleep duration patterns had with a gold standard assessment of caloric intake, objective assessments of physical activity and body fat, and BMI based on measured weight and height. It was expected that sleep duration trajectories indicative of habitual short sleep or the development of such patterns, would be related to higher daily caloric intake, less daily physical activity, and increases in BMI and percent body fat. The expectation that multiple sleep duration trajectories would emerge was not observed and, therefore, the hypotheses related to the expected trajectories were not supported. This finding is in opposition to existing literature suggesting that in larger samples and population studies there are distinct sleep duration trajectories in children, adolescents, and adults with those trajectories indicating shorter sleep duration across time associated with higher BMI [24–28].

Upon exploring the data further through within and between person effects, it was observed that only subjective sleep quality emerged as a key component. Specifically, subjective sleep quality was observed to predict changes in BMI over time in both a direct and within person effect. Therefore, as subjective sleep quality decreased, BMI increased across the sample and within individuals. This pattern was not observed for the objective measure of sleep duration, potentially highlighting the role that the other domains of sleep health may play in obesity risk. This finding contributes to the existing literature suggesting an association between subjective sleep quality and BMI, such that poor sleep quality is related to overweight and obesity [14, 29–32]. However, it is important to note that the existing literature on subjective sleep quality and BMI both cross-sectionally and longitudinally has focused on individuals who were overweight and obese. The current sample, on average, had a BMI < 25 at all three time points indicating normal weight status. Further, depending on what cut-off is used, as the cut-off for good sleep quality for the general population is < 5 [22] and for college students is < 8 [33], the current sample would generally be categorized as good quality sleepers. Therefore, the fact that the sample would be considered good quality sleepers, normal weight, and on average sleeping more than 7 hours per night may have been a limiting factor for observing sleep trajectories or relationships with the other weight gain risk behaviors.

Although Project XXXXXXX was designed to address limitations within the literature, the current study is not without its own limitations. Sample characteristics are the primary limitations of the study. In addition to being a primarily White, Female sample, the sample was also on average of normal weight status, healthy, had good sleep health, and consumed less than 2,000 calories per day. This does limit generalizability and limits the comparison to the existing literature on different sample characteristics. Further, this may indicate that we did not recruit a sample that is at risk for the development of overweight or obesity based on these characteristics or related protective factors that were not assessed. Additionally, the sample may not be representative of a conventional college sample as more than half lived off campus and almost half of the sample lived off campus with their parents. More than half the sample was also employed and living off campus was related to having employment (r = .47, p < .001). While employment may not be unconventional for college students, these two characteristics taken together does suggest that a large proportion of the sample were outside the college environment more, which may impact sleep schedules, sleep quality, and food choices and options. Lastly, although designed to reduce participant burden, a one-week data collection window for each time point may have been too short to capture more robust or nuanced data as well.

In conclusion, the findings of the current study continue to suggest that sleep health may play an important role related to changes in factors associated with the development of obesity. The non-significant findings should not be taken to mean that sleep duration and quality do not impact obesity risk factors, such as food intake, physical activity, or metabolic alterations. Instead, the findings do suggest that characteristics of the current sample may have served as protective factors for obesity risk and that additional research needs to be done to determine what characteristics and factors do contribute to obesity risk as it relates to poor sleep.

This study was approved by the Institutional Review Board at XXXXXXX (IRB# XXXXXXX) and is registered as a longitudinal observational study on clinicaltrials.gov (XXXXXXX). All procedures performed were in accordance with the ethical standards of the institutional and/or national research committee and with the 1964 Helsinki declaration and its later amendments or comparable ethical standards. Potential participants were asked to read the informed consent form in full and their questions were answered by a research assistant. They signed two copies of the informed consent form (one was taken home) if they were interested in going forward with participating.

## Analytic Plan and Results

The present study utilizes clustered longitudinal data. The highest cluster level, level 1, is represented by the participants (N = 115) and level 2 is represented by scores clustered within each participant. Specifically, at level 2 there are six scores clustered within each participant. The six scores are the sleep and BMI scores at Times 1, 2, and 3. A Principal Components Analysis (PCA) was used to examine hypothesis 1. Preparing the data for a PCA included calculating derivatives of the vectors, centering using center reduction methodology, and normalizing the data. This method was employed to determine whether there was adequate separation between groups to create proper trajectories. One hundred and fourteen predictor vectors of length 6 were included for this residual PCA with eigenvalue-eigenvector sorting, and 95% significance. Variance-covariance methodology was also employed for the PCA. The PCA suggested between 6 and 11 groups of conflicting trajectories with the largest group consisting of participants with missing data. To verify these findings, silhouetting (k-mean clustering) was performed with elbow methodology to find the inflection point of significance. Given the large number of groups, coupled with the small number of participants per group, the trajectories were not statistically significant.

A series of linear mixed effects models were performed using the following packages within the statistical program R: lme4, tidyverse, and lubridate. These analyses were conducted to better understand the non-significant trajectory results and have the goal of disaggregating the within and between person effects [23]. Participants still served as the level 1, with scores clustered within themselves at level 2. Predictor variables included objective sleep duration (per the Actiwatch), and the PSQI at each time point. Person mean scores for each predictor were calculated and turned into a new variable; followed by a mean centered version of each time point score (i.e., Time1 variable - Person mean). The centering function in R was utilized to center the final variables for analysis. Outcome variables included BMI (per measured weight and height), total body fat percent (per the DXA), minutes of moderate physical activity (per the Actigraph), minutes of vigorous physical activity (per the Actigraph), and kcal consumption (per the ASA-24). Three separate models were run for each outcome to predict sleep variables onto weight-related variables; one model included the direct predictors, the second model included the person mean versions of the predictors, and the third model included the person mean centered versions. A total of 9 models were conducted with 115 participants in accordance with Curran & Bauer [23]. These unique analyses parse out the different effects simultaneously happening within the data through three direct models, three between-person models, and three within-person models. The first three models aimed to find any direct correlations between the predictor variables and the respective outcome. The next three models utilize the person means of their respective variables across time, which demonstrates a between persons comparison of the predictors on the outcome. The final three models utilize the person mean centered variable which show within person changes across time. For BMI there were two significant predictors. First, PSQI scores (where higher scores indicate worse sleep quality) were found to be positively related to BMI (estimate = 0.046, SE = 0.011, t = 4.12). Secondly, mean-centered PSQI scores were found to be positively related to BMI (estimate = 0.049, SE = 0.011, t = 4.44). There were no other significant effects found within these analyses.

## Figures and Tables

**Table 1:** Demographic Characteristics

Characteristic	n	%
Total sample size	115	(100)
Age		
17	8	(7.0)
18	101	(87.8)
19	6	(5.2)
Sex		
Female	81	(70.4)
Male	34	(29.6)
Ethnicity		
Hispanic or Latino	6	(5.2)
Not Hispanic or Latino	105	(91.3)
Chose not to answer	4	(3.5)
Race		
African American or Black	19	(16.5)
American Indian or Alaskan		
Native	1	(.9)
Asian	7	(6.1)
Middle Eastern	5	(4.4)
White	81	(70.4)
Chose not to answer	2	(1.7)
Current residence		
On campus	45	(39.1)
Off-campus with parent	54	(47.0)
Off-campus without parent	15	(13.0)
Chose not to answer	1	(.9)
Current employment stams		
Employed	62	(54.0)
Not employed	53	(46)
Currently trying to lose weight		
Yes	30	(26.1)
No	85	(73.9)

## Data Availability

Data can be accessed from the primary author.
